# Motor contributions to the temporal precision of auditory attention

**DOI:** 10.1038/ncomms6255

**Published:** 2014-10-15

**Authors:** Benjamin Morillon, Charles E. Schroeder, Valentin Wyart

**Affiliations:** 1 Department of Psychiatry, Columbia University Medical Center, New York, New York 10032, USA; 2 Cognitive Neuroscience and Schizophrenia Program, Nathan Kline Institute, Orangeburg, New York 10962, USA; 3 Département d’Etudes Cognitives, Laboratoire de Neurosciences Cognitives, Inserm unit 960, Ecole Normale Supérieure, Paris 75005, France

## Abstract

In temporal—or dynamic—attending theory, it is proposed that motor activity helps to synchronize temporal fluctuations of attention with the timing of events in a task-relevant stream, thus facilitating sensory selection. Here we develop a mechanistic behavioural account for this theory by asking human participants to track a slow reference beat, by noiseless finger pressing, while extracting auditory target tones delivered on-beat and interleaved with distractors. We find that overt rhythmic motor activity improves the segmentation of auditory information by enhancing sensitivity to target tones while actively suppressing distractor tones. This effect is triggered by cyclic fluctuations in sensory gain locked to individual motor acts, scales parametrically with the temporal predictability of sensory events and depends on the temporal alignment between motor and attention fluctuations. Together, these findings reveal how top-down influences associated with a rhythmic motor routine sharpen sensory representations, enacting auditory ‘active sensing’.

## Abstract (web summary)

Motor activities, such as rhythmic movements, are implicated in regulating attention. Here, the authors find that rhythmic movements sharpen the temporal selection of auditory stimuli by facilitating the perception of relevant stimuli, while actively suppressing the interference from irrelevant stimuli.

## Graphical abstract

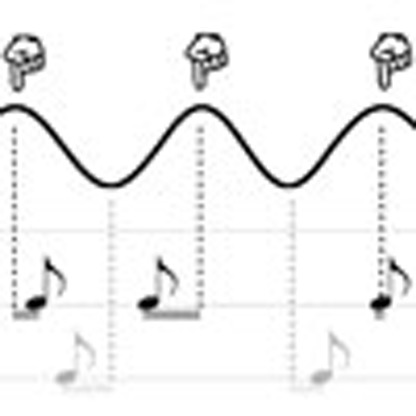

In neurobiology, the term ‘active sensing’ acknowledges the fact that animals generally gather information from the environment using motor sampling routines^1^,^2^,^3^,^4^,^5^,^6^,^7^. In addition to controlling the orienting of sensing organs (for example, ocular saccades, tactile and haptic exploration, whisking or sniffing), the motor system modulates the processing of sensory information via top-down corollary discharge signals (that is, copies of effector commands sent to sensory structures^1^), which effectively align sensory processing in phase with ongoing motor sequences.

Rhythm is a prominent characteristic of motor activity, particularly evident in walking/running and vocalizing, and is reflected in important motor sampling patterns, such as sniffing^8^ and whisking^9^ in rodents and visual search in primates^10^. Rhythmic sampling has been described as periodic fluctuations in attention that modulate the gain of sensory inputs that form the basis of perception and is referred to as temporal—or dynamic—attending^11^,^12^. This noncontinuous extraction of sensory information capitalizes on the fact that many natural stimuli are also organized in rhythmic or quasi-rhythmic streams. By learning the resulting temporal regularities, the brain is able to predict the occurrence of future sensory events and optimize their processing^13^,^14^,^15^. As such, attention is the essential component of active sensing^4^. While attention can operate in the absence of any overt motor activity, as in covert spatial attention^16^, motor and/or premotor structures are almost invariably recruited during temporal attending^13^,^17^,^18^. This points to a deep, potentially causal, relationship between the motor system and attention^19^.

In the present study, we investigated the role of the motor system in temporal attention by quantifying its distinct and unique influence on sensory processing, in an active sensing paradigm. We chose the auditory modality because, in contrast to the other senses, there exists no simple causal relationship between bottom-up sensory input and an overt motor sampling routine. In natural listening, humans use smooth motor routines (for example, orienting the head) to enhance binaural sound localization^20^; however, they do not typically use movements to trigger phasic volleys of bottom-up auditory input as they would when exploring scenes or surfaces with the eyes or hands, or as rodents do in whisking and sniffing^2^,^6^. However, in rhythmic contexts, a strong relationship forms between auditory and motor systems^21^,^22^,^23^,^24^,^25^, and this latter is a key structure for the precise estimation of (short) durations^26^,^27^. A few recent studies have shown that phasic movements (for example, tapping or moving the head to a beat) can enhance rhythm perception^28^,^29^,^30^,^31^,^32^,^33^; however, they have controlled variably for the bottom-up auditory stimulation generated by these movements. More importantly, to our knowledge, no study to date has examined the specific information and timing mechanisms that the motor system may use to facilitate temporal attending.

To test whether motor activity helps to synchronize temporal fluctuations of attention with the timing of events in a task-relevant stream, we develop a paradigm to behaviourally quantify the precision of temporal attention during auditory perception. In three interrelated behavioural experiments, we isolate an active motor influence on the temporal precision of auditory attention. We show that this rhythmic top-down process requires an exquisite temporal alignment between motor, attentional and auditory signals.

## Results

### Motor contributions to temporal attention

**Figure 1:**
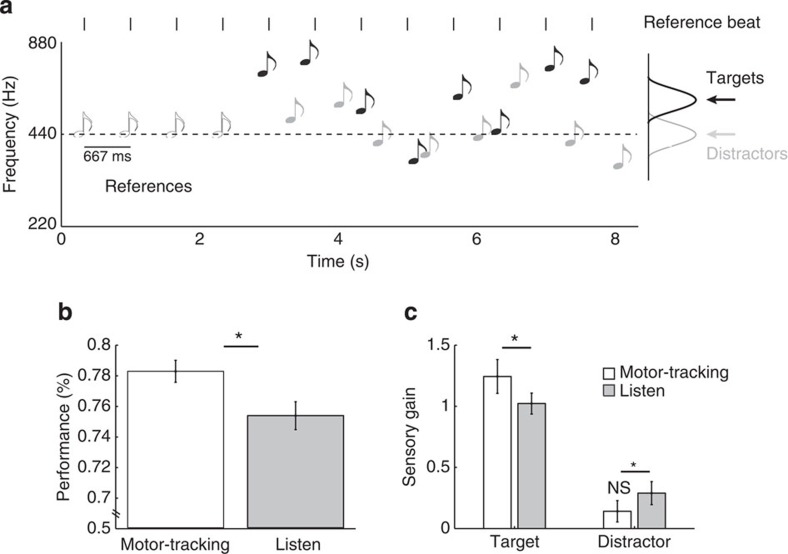
Experimental design and main effect of motor tracking. Experiment 1: (**a**) Rhythmic sequences of 20 pure tones were presented binaurally on each trial. Four reference tones preceded an alternation of eight target and eight distractor tones of variable frequencies. Targets occurred in phase with the preceding references, whereas distractors occurred in antiphase. Participants had to decide whether the mean frequency of targets was higher or lower than the reference frequency. In the listen condition, participants performed the task without moving before the end of the sequence. In the motor-tracking condition, participants performed the task while expressing the reference beat by moving their index finger. (**b**) Average categorization performance in the motor-tracking and listen conditions. (**c**) Contributions of targets and distractors to the decision in the motor-tracking (white bars) and listen (grey bars) conditions. Sensory gains were estimated for each target and distractor tone using a multivariate logistic regression of choice against a weighted sum of the information provided by each tone, expressed in relative distance from the reference frequency. Sensory gains were pooled separately across targets and distractors. Error bars indicate s.e.m. Stars/NS indicate significant/nonsignificant differences (*n*=21; paired *t*-tests or *t*-tests against zero; *P<0.05).

In all three experiments, we asked the participants to categorize sequences of pure tones as higher- or lower-pitched, on average, than a reference frequency *f*_0_. In order to drive rhythmic fluctuations in attention, we presented the tones (targets) in phase with a reference beat, and in antiphase with irrelevant, yet physically indistinguishable tones (distractors). Each trial started with the rhythmic presentation of four ‘reference’ tones indicating both the reference frequency (*f*_0_) and beat. They were followed by the alternation of eight targets and eight (or nine) distractors (see Methods) of variable frequencies, respectively, presented in quasi phase and antiphase with the reference beat (Fig. 1a). This interleaved delivery of sensory events forced the participants to use the reference beat to discriminate between targets and distractors—that is, to maximize the integration of relevant sensory cues (targets) while minimizing the interference from irrelevant ones (distractors). This protocol ensured that their attentional focus was temporally modulated over an extended time period.

The participants performed a two-alternative pitch categorization task at the end of each trial, by deciding whether the mean frequency of targets *f*^tar^ was higher or lower than *f*_0_. The mean frequency of distractors *f*^dis^ was always equal to *f*_0_, hence noninformative. The absolute distance between *f*^tar^ and *f*_0_ was titrated for each participant before the experiment to reach threshold performance (see Methods). The task consisted of two conditions: in the ‘listen’ condition, participants performed the task while staying completely still during the duration of the trial; in the ‘motor-tracking’ condition, they performed the task while pressing rhythmically a noiseless, pressure-sensitive pad with their index finger in phase with the reference beat. Therefore, the single difference between the two conditions was that participants moved their finger in rhythm with the relevant sensory cues in the motor-tracking condition. While it is not possible to control for the covert involvement of motor and/or premotor structures during temporal attending tasks, the comparison between listen and motor tracking conditions allowed us to quantify the influence of overt (relative to covert) motor activity on the precision of temporal attention—that is, the ability of the participants to make selective use of targets, not distractors, in their subsequent decision.

### Motor tracking improves target-distractor segregation

In a first experiment, the comparison between listen and motor-tracking conditions revealed a significant increase in categorization performance during overt motor tracking (paired *t*-test, *t*_20_=2.3, *P*<0.05; Fig. 1b). To characterize this net effect, we quantified the relative contribution of each tone to the decision. We estimated the sensory ‘gain’, in other words the additive contribution, assigned to each target (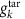) and distractor (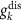), to the subsequent decision (higher- or lower-pitched than *f*_0_). We calculated these parameters across trials via a multivariate logistic regression of choice on the basis of a linear combination of the frequencies of the sixteen tones (eight targets and eight distractors, see Methods):




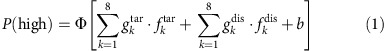




where *P* (high) is the probability of judging the target sequence as higher pitched, Φ[.] the cumulative normal density function, 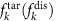 the frequency of the target (distractor) tone at position *k* in the sequence (expressed in logarithmic distance to *f*_0_) and *b* an additive response bias towards one of the two choices. We first observed that, across conditions, participants were able to assign greater gain to targets than distractors in their decision (repeated-measures analysis of variance (ANOVA), F_1,20_=39.0, *P*<0.001; Fig. 1c and Supplementary Fig. 1a). Moreover, as predicted by the logistic regression model, the influence a given target wielded on the subsequent decision scaled parametrically with its absolute distance from *f*_0_, whereas this effect was effectively absent for distractors (Supplementary Fig. 1b). In other words, in both conditions, participants were able to focus their temporal attention to segregate targets from distractors and increase the selectivity of their subsequent decision.

Interestingly, for both targets and distractors, we observed significant changes in sensory gain between listen and motor-tracking conditions, in opposite directions (repeated-measures ANOVA, interaction: F_1,20_=6.9, *P*<0.05): the contribution of targets increased in the motor-tracking condition (F_1,20_=5.8, *P*<0.05), whereas the contribution of distractors decreased (F_1,20_=4.6, *P*<0.05). While distractors interfered significantly with decision-making in the listen condition (*t*-test against zero, *t*_20_=3.1, *P*<0.01), this was not the case in the motor-tracking condition (*t*_20_=1.6, *P*=0.12). These results indicate that the increased categorization performance observed in the motor-tracking condition is because of an improved temporal segregation between sensory cues—that is, the joint influence of an increased sensitivity to targets and a decreased sensitivity to distractors.

### Motor tracking drives rhythmic fluctuations in attention

**Figure 2:**
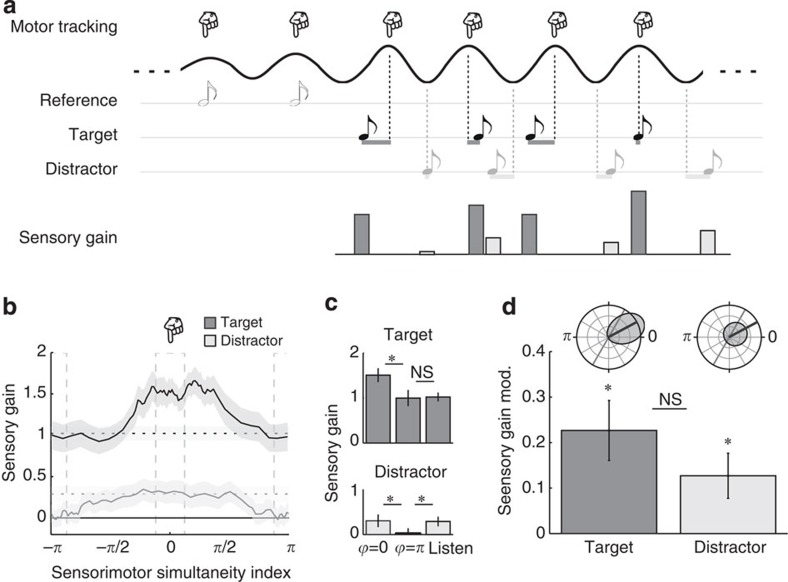
Motor-tracking-locked rhythmic gain model. (**a**) Description of the model. First row: rhythmic motor tracking in phase with the reference beat throughout the sequence. Second row: references. Third row: targets presented in phase with the reference beat. Dark grey lines indicate the temporal distance between the motor act and the onset of the target. Fourth row: distractors presented in antiphase with the reference beat. Light grey lines indicate the temporal distance between the motor act and the onset of the distractor. Fifth row: gains assigned to successive targets and distractors. (**b**–**d**) Experimental validation of the model. (**b**) Target/distractor gains sorted according to their temporal distance to motor acts (SSI; dashed lines correspond to the listen condition). (**c**) Detail of the model-predicted best (*ϕ*=0) and worst (*ϕ*=*π*) octile, and comparison with the listen condition. (**d**) Upper panel: between-subject distributions of SSI for which the gain is maximally modulated (0: in-phase, *π:* antiphase). Lower panel: behavioral variability explained by taking SSI into account. (Shaded) error bars indicate s.e.m. Stars/NS indicate significant/nonsignificant differences (*n*=21; paired *t*-tests or *t*-tests against zero; *P<0.05).

We hypothesized that a simple model in which motor tracking drives rhythmic fluctuations in temporal attention could account for the observed findings (Fig. 2a). Such a model makes two key predictions in our experiment: (1) a negative relationship between the effects of motor tracking on the sensory gain of targets and distractors and (2) a parametric modulation of the sensory gain assigned to each tone by its degree of simultaneity with the closest motor act. As a prerequisite, we verified that participants were tracking the reference beat (provided by the first four reference tones) rather than individual tones in the motor-tracking condition. The timing of individual motor acts was indeed better synchronized to the reference beat than to the onset of the slightly jittered targets or distractors (paired *t*-tests, both *t*_20_>12.0, *P*<0.001; Supplementary Fig. 2a,b).

We then estimated, across tested participants, the degree of correlation between the effects of motor tracking (by contrasting motor-tracking and listen conditions) on the sensory gain of targets and distractors, and found it to be significantly negative as predicted by the model (linear regression, *r*=−0.50, d.f.=19, *P*<0.05). In the motor-tracking condition, we then sorted targets and distractors as a function of their degree of simultaneity with the closest motor act (sensorimotor simultaneity index, or SSI), as estimated by their phase in a reconstructed motor oscillation (Fig. 2a,b, see Methods). We quantified the effect of sensorimotor simultaneity on sensory gain by comparing estimates obtained for the first (most synchronous) and last (most asynchronous) octiles of SSI. As predicted by the model, we observed a significant modulation of the sensory gain assigned to each target and distractor as a function of SSI (paired *t*-test, targets: *t*_20_=3.2, *P*<0.005, distractors: *t*_20_=2.5, *P*<0.05; Fig. 2c). The comparison between sensory gains obtained in the listen and motor-tracking conditions further indicated that motor tracking was always beneficial to performance. Indeed, targets occurring in synchrony with motor acts exhibited an increased sensory gain relative to the listen condition (paired *t*-test, *t*_20_=4.3, *P*<0.001), whereas distractors did not (*t*_20_=0.1, *P*>0.5). Conversely, distractors occurring in phase opposition to motor acts exhibited a reduced sensory gain (*t*_20_=2.6, *P*<0.05), whereas targets did not (*t*_20_=0.2, *P*>0.5). These results show that the influence of motor activity on sensory processing, as estimated in the motor-tracking condition, adds *positively* to the effect of temporal attention, as estimated in the listen condition.

For completeness, we quantified this parametric effect of SSI on sensory gain by estimating the modulation strength associated with each target (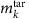) and distractor (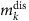) tone. For this purpose, we re-fitted the multivariate logistic regression model described above (equation 1), with the addition of a multiplicative interaction term cos (SSI_*k*_)·*f*_*k*_ to each target and distractor tone, corresponding to the cosine of the SSI—that is, from perfect synchrony (+1) to perfect phase opposition (−1):




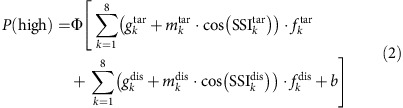




This analysis confirmed that the gains of both targets (*t*-test against zero, *t*_20_=3.4, *P*<0.005) and distractors (*t*_20_=2.6, *P*<0.05) were significantly modulated by sensorimotor simultaneity, and to a comparable extent (paired *t*-test, *t*_20_=1.6, *P*=0.14; Fig. 2d). Note that the sensorimotor phase corresponding to the maximal sensory gain did not differ between targets and distractors, as predicted by the model (parametric Watson–Williams test, F_1,20_=0.1, *P*>0.5). As a control, we conducted the same analysis using the degree of simultaneity between each tone and the theoretical reference beats (instead of the recorded motor acts) to assess whether the observed gain modulation could be attributed to a nonmotor influence. Compared with the original analysis, this nonmotor simultaneity index yielded weaker modulations for both targets and distractors (repeated-measures ANOVA, F_1,20_=10.9, *P*<0.005; Supplementary Fig. 2c). In fact, this control analysis revealed no significant gain modulation in either condition (*t*-tests against zero, both *t*_20_<1.4, *P*>0.1). This last result indicates that the modulation of sensory gain by sensorimotor simultaneity quantifies selectively the influence of motor activity on sensory processing.

### Motor contributions scale with temporal predictability

In a second experiment, we investigated the role of stimulus and motor rhythmicity on the motor-tracking effect by varying the precision of sensorimotor simultaneity—which depends both on the extrinsic (experimenter-controlled) rhythmicity of the auditory sequences and on the intrinsic (participant-controlled) rhythmicity of motor tracking. Taking advantage of our ability to control stimulus rhythmicity, we manipulated independently the jittering of targets and distractors using rhythmic (non-jittered) or jittered (67 ms) tone sequences. This yielded four conditions of varying degrees of predictability: fully rhythmic (non-jittered), semirhythmic (jittered targets or distractors) or arrhythmic (jittered targets and distractors).

**Figure 3:**
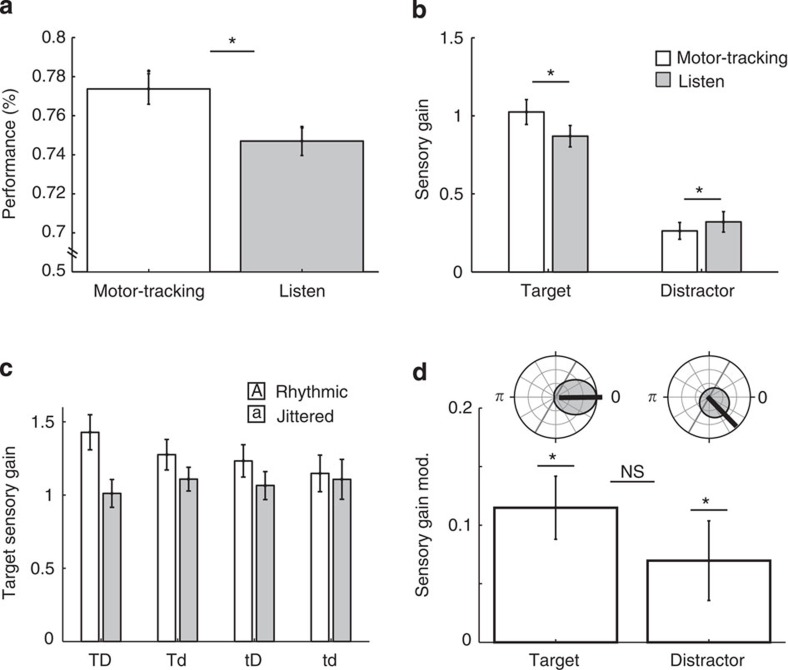
Influence of acoustic rhythmicity. Experiment 2: four references preceded an alternation of nine distractors and eight targets. Target and/or distractor sequences were either presented in a rhythmic or jittered manner. As in experiment 1, the task was divided into listen and motor-tracking conditions. (**a**) Average categorization performance in the motor-tracking and listen conditions, averaged across the different rhythmicity conditions. (**b**) Contributions of targets and distractors to the decision in the motor-tracking (white bars) and listen (grey bars) conditions, averaged across the different rhythmicity conditions. (**c**) Contribution of targets to the decision, detailed for each condition. T/t indicates target rhythmic/jittered conditions, D/d distractor rhythmic/jittered conditions and white/grey bars motor-tracking/listen conditions. (**d**) Additional results in the motor-tracking condition, averaged across the different rhythmicity conditions. Upper panel: angle histograms of SSI at which the gain is maximally modulated (0: in-phase, *π*: antiphase). Lower panel: behavioral variability explained by taking SSI into account. (Shaded) error bars indicate s.e.m. Stars/NS indicate significant/nonsignificant differences (*n*=18; paired *t*-tests or *t*-tests against zero; *P<0.05).

We first replicated the main findings of Experiment 1: an ANOVA revealed only a main effect of motor tracking on categorization performance (repeated-measures ANOVA, F_1,17_=6.3, *P*<0.05; Fig. 3a). The performance increase observed during motor tracking corresponded again to a higher gain for targets (F_1,17_=9.9, *P*<0.01) and a lower gain for distractors (F_1,17_=4.4, *P*=0.052; Fig. 3b). In full accordance with Experiment 1, the gain assigned to both targets and distractors was modulated by sensorimotor simultaneity in the motor-tracking condition (*t*-test against zero, targets: *t*_17_=4.3, *P*<0.001, distractors: *t*_17_=2.1, *P*=0.056) and to a comparable extent (paired *t*-test, *t*_17_=1.3, *P*=0.2; Fig. 3d).

Second, as expected, we observed that the jittering of targets and distractors influenced the precision of motor tracking (repeated-measures ANOVA, target jitter: F_1,17_=115.0, *P*<0.001, distractor jitter: F_1,17_=78.0, *P*<0.001; Supplementary Fig. 3). This indicates that degrading the extrinsic rhythmicity of auditory sequences altered the intrinsic rhythmicity of motor tracking, thereby confirming that the active sensorimotor circuit operates as a loop. Moreover critically, the jittering of targets and distractors also influenced the motor-tracking effect on the sensory gain of targets (target jitter: F_1,17_=5.3, *P*<0.05, distractor jitter: F_1,17_=6.6, *P*<0.05; Fig. 3c). This result indicates that the motor contribution to the selection of relevant sensory cues depends on the temporal predictability of the sensory cues: the higher the predictability, the stronger the beneficial effect of motor tracking on the attentional selection of targets among distractors.

### Motor contributions depend on synchrony with attention

In a third experiment, we tested whether the beneficial influence of motor activity on sensory processing relies on co-occurring fluctuations in temporal attention. More precisely, we asked whether the enhancement of sensory gain during motor tracking depends critically on the precise temporal relationship between motor activity and the focus of temporal attention. We contrasted three alternative hypotheses: (1) each motor act is always accompanied by a transient enhancement of sensory gain (motor-driven enhancement), (2) the precision of temporal attention always benefits from overt motor activity, irrespectively of their precise phase relationship (attention-driven enhancement) or (3) motor activity and temporal attention must fluctuate in synchrony for a beneficial effect to occur (synergistic enhancement). To arbitrate among these three hypotheses, we recorded behavioural data from a variant of Experiment 1 (Fig. 1) in which the tones previously labelled as targets were now labelled as distractors, and *vice versa*. Targets thus occurred in antiphase with the reference beat. Despite this change, we kept the same motor-tracking instruction as in Experiment 1—namely, to follow the reference beat. This condition imposed a constant phase opposition between motor and attention fluctuations: participants had to pay attention to sensory events off-beat while finger-pressing on-beat.

**Figure 4:**
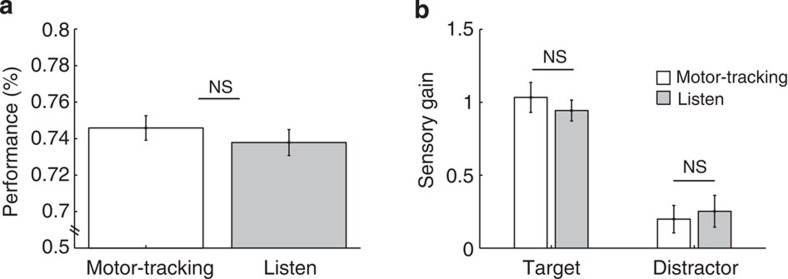
Influence of attention-motor coupling. Experiment 3: in contrast to experiment 1, distractors occurred in phase with the preceding reference tones (and motor acts), whereas targets occurred in antiphase with the reference tones. This design allowed to temporally dissociate motor tracking and temporal attention: participants pressed their index finger in phase with distractors while paying attention to targets. (**a**) Average categorization performance in the motor-tracking and listen conditions. (**b**) Contributions of targets and distractors to the decision in the motor-tracking (white bars) and listen (grey bars) conditions. Error bars indicate s.e.m. NS indicates nonsignificant differences (*n*=21; paired *t*-tests; *P<0.05).

In terms of difference between motor-tracking and listen conditions, a motor-driven enhancement predicts that motor tracking in phase with distractors should impair categorization performance when compared with the listen condition. By contrast, an attention-driven enhancement predicts that motor tracking in phase with targets (Experiment 1) and distractors (Experiment 3) should be equally beneficial to performance. Finally, a synergistic enhancement predicts that motor tracking in phase with distractors should not have any effect on performance. In accordance with the latter hypothesis, we failed to observe any significant difference between motor-tracking and listen conditions (Fig. 4) either in terms of accuracy (paired *t*-test, *t*_20_=0.8, *P*>0.4), sensory gain of targets (*t*_20_=1.4, *P*>0.1) or distractors (*t*_20_=−0.8, *P*>0.2). A direct comparison of Experiments 1 and 3, however, yielded no significant difference between the observed effect sizes (mixed-design ANOVA, accuracy: F_1,40_=1.8, *P*=0.19, sensory gain of targets: F_1,40_=1.4, *P*>0.2, sensory gain of distractors: F_1,40_=1.0, *P*>0.2).

To obtain conclusive evidence, we estimated and compared the modulation of sensory gain by sensorimotor simultaneity (averaged over targets and distractors) during motor tracking in phase with targets (Experiment 1) and distractors (Experiment 3). A motor-driven enhancement predicts that the sensorimotor phase corresponding to the maximal sensory gain should be identical (and close to zero-lag) whether motor tracking occurs in phase with targets (Experiment 1) or distractors (Experiment 3). By contrast, an attention-driven enhancement predicts that the maximal sensory gain should occur for opposite sensorimotor phases in the two experiments. Last, a synergistic enhancement predicts no modulation of sensory gain when motor tracking occurs out-of-phase with attention—that is, in phase with distractors (Experiment 3). As suggested by the lack of motor-tracking effect on categorization performance, sensorimotor simultaneity did not modulate sensory gain in Experiment 3 (*t*-test against zero, *t*_20_=0.1, *P*>0.5). A direct comparison between the two experiments revealed that the modulation of sensory gain observed in Experiment 1 (Fig. 2d) was significantly suppressed in Experiment 3 (two-sample *t*-test, *t*_40_=2.7, *P*=0.01), thereby ruling out formally a purely motor-driven enhancement of sensory gain. Furthermore, the average modulation strength measured in Experiment 3 was also significantly weaker than the opposite of the value obtained in Experiment 1 (*t*_40_=2.6, *P*=0.01), in strong disagreement with the 180° shift in preferred phase predicted by a purely attention-driven enhancement of sensory gain.

To provide positive evidence in favour of a synergistic enhancement of sensory gain by temporal attention and motor activity, we tested whether the nonsignificant modulation of sensory gain observed in Experiment 3 was likely because of a genuine absence of effect (rather than a lack of statistical sensitivity). For this purpose, we computed the Bayes Factor associated with the corresponding effect under the same parametric assumptions as conventional statistics (see Methods for more details). We obtained a Bayes Factor of 0.30 (less than 1/3), indicative of the absence of effect predicted by the synergistic hypothesis.

Together, this pattern of findings indicates that the differences observed between the two experiments are subtle—that is, not captured by comparisons between motor-tracking and listen conditions in terms of aggregate measures such as categorization performance or average sensory gain. Considering the fluctuations in temporal synchrony between sensory cues and individual motor acts, however, suggested that the sensory gain modulation observed during motor tracking in phase with targets was entirely absent during motor tracking in phase with distractors. This difference suggests that motor rhythms require synchronous fluctuations in attention to enhance sensory processing, thereby supporting a synergistic enhancement of sensory processing that relies on the temporal alignment between motor and attention fluctuations.

## Discussion

Our findings show that rhythmic movements engage a top-down modulation that sharpens the temporal selection of auditory information, facilitating perception of relevant items and suppressing perception of irrelevant items. Our results also confirm that, while top-down influences from the motor system are often conflated with bottom-up ones, they can play a distinct and fundamental role in sensory processing. Recent behavioural experiments on the top-down role of the motor system in auditory perception show that motor activity can reset the perceptual organization of an auditory scene^20^, facilitate pulse extraction^28^,^29^,^30^,^32^,^33^ and strengthen attention to auditory stimuli^24^,^31^.

Here we went several important steps further by firmly isolating top-down motor influences on auditory stimulus processing, and by showing that the engagement of the motor system improves: (1) the precision of temporal attention and, thus, (2) the quality of sensory selection. Overt motor rhythms improve the temporal segmentation of auditory information, which results in a stronger multiplicative gain assigned to decision-relevant information and a more efficient suppression of distractors. Importantly, we found that the modulation has a cyclic profile, since the processing of both targets and distractors was modulated, in opposite directions (Fig. 1). The increased inhibition of distractors expands on dynamic attending theory^12^, which does not predict suppression in distractor processing below a no-movement baseline. The importance of our findings is underscored by recent reports of periodic fluctuations in attention during nonrhythmic spatial tasks, which suggest that attention is rhythmic in essence^15^,^34^,^35^,^36^,^37^. Altogether, these results are consistent with recent electrophysiological studies showing that slow cortical oscillations, which can act as instruments of sensory selection by modulating the excitability of task-relevant neuronal populations^15^, are the substrate of temporal attention^17^,^38^,^39^,^40^ and are found in the primary motor cortex to co-vary with the occurrence of temporally predictable task-relevant cues^41^.

To further characterize the mechanisms underlying this motor-driven modulation of sensory gain, we studied how the quality of sensory processing depended on the temporal relationship between motor and auditory systems (Experiment 2), and motor and attention systems (Experiment 3). We derived a SSI, a measure exploiting both the intrinsic (Fig. 2 and Supplementary Fig. 3) and extrinsic (Fig. 3) sources of sensorimotor variability, and showed that the motor-related enhancement of auditory segmentation scaled parametrically with this factor. Importantly, SSI reflects the quality of the temporal prediction made by the motor system. The activity in the lateral premotor cortex and its connectivity with auditory cortices are known to co-vary with the temporal predictability of environmental events^42^,^43^. Under an active sensing account, the motor system directs sensing organs towards relevant stimuli while generating top-down corollary discharge signals^1^. This, respectively, structures the content of sensory information inflow while predictively modulating sensory processing according to the temporal and spatial patterns of motor activity patterns, thus providing ‘when’ and ‘where’ predictions at a minimum^44^. Our results thus reinforce the idea that the motor system can be viewed as a predictive system, generating putative beats and getting engaged in the analysis of temporal sequences^4^,^5^,^45^,^46^,^47^. In addition, we found that the motor-related improvement depended on the temporal predictability of targets and distractors in an additive and comparable way (Fig. 3). This result speaks directly to the question of whether target enhancement and distractor suppression are mediated by distinct mechanisms, or by a single one^48^. Our findings and proposed model concur with both the normalization model of attention^49^ and the spectrotemporal filter mechanism^39^, in positing a single mechanism for target enhancement and distractor suppression.

The temporal alignment between motor rhythms and temporal attention proved also to be a key component of this paradigm. Indeed, we failed to observe a significant modulation of auditory segmentation by motor-related activity when motor tracking took place in phase with distractors and in phase opposition to targets (Fig. 4 and Supplementary Fig. 4). This suggests that top-down motor corollary discharge signals alone do not countermand those of temporal attention. If temporal attention can thus operate independently of the motor system, premotor cortical activations observed in studies of temporal attention^13^,^18^,^23^ may be coding other task parameters, such as the predictability of the sequence. The requirement for temporal alignment also confirms that attention is the single crucial component of active sensing^4^. Finally, this result also mirrors the recent dissociation between predictive and attentional influences on visual perception^50^,^51^.

It merits emphasis that, in the three experiments reported here, we never observed deleterious effects of motor tracking on the selection of sensory information: either when the prediction made by the motor system was inaccurate (large SSI) or when motor tracking occurred in phase with distractors. It thus seems that the motor-related improvement of sensory selection is conditional on the synchrony of individual movements with the augmenting phase of fluctuations in temporal attention: in other words, prediction-weighted motor rhythms enhance sensory processing only when they reinforce gain enhancement triggered by attention. Moreover, the motor-tracking effect is additive with that of (covert) temporal attention, as indexed by the listen condition.

On a broader level, our results confirm the operation of active sensing in the auditory domain, with motor rhythms representing substrates of temporal prediction, and they clarify the role of foot tapping and head nodding during music playing^21^. It appears that a synergistic interaction operates between auditory, motor and attention systems during sampling of auditory information^52^. While bottom-up audio-motor message passing allows the build-up of temporal prediction, its top-down counterpart drives fluctuations in sensory gain (possibly via the frontoparietal dorsal attention network). These two mechanisms are dependent on exogenous and endogenous attention mechanisms, respectively^53^ and, while their neurophysiological bases are not yet certain, plausible substrates for multiplexing of top-down and bottom-up activities have been identified^54^,^55^. Critically, active sensing appears to be conditional upon the in-phase alignment of attention and motor rhythms. The observation of additive effects of motor activity and temporal attention suggests that closely interrelated but dissociable neural circuits contribute to drive motor prediction- and attention-related influences, and reinforce the view that prediction and attention have to be considered as distinct sources of top-down influences in future research^50^.

## Methods

### Participants

In all, 21, 18 and 21 healthy adult participants were, respectively, recruited for Experiments 1, 2 and 3 (age range: 20–59 years; 54% of females). The experiment followed the local ethics guidelines, and informed consent was obtained from all participants before the experiment. All had normal audition and reported no history of neurological or psychiatric disorder. In order to obtain results that generalize broadly, we did not select participants based on musical training.

### Stimuli

Auditory stimuli were sampled at 44,100 Hz and presented binaurally via headphones (equipped with a noise-reduction system) at a comfortable hearing level, using the Psychophysics-3 toolbox^56^ and additional custom scripts written for MATLAB (The Mathworks). Each trial consisted of a sequence of pure tones presented at an average rate of 1.5 Hz (interstimulus interval (ISI)=667 ms). Each pure tone lasted 100 ms with a dampening length and attenuation of 10 ms and 40 dB, respectively. Pure tones were qualified as references (first four tones), targets and distractors (Fig. 1a).

### Experimental design

All three experiments had the same general design: each trial was composed of rhythmic sequences of 20 pure tones (Fig. 1a). They were initiated by four reference tones indicating both the reference frequency (*f*_0_=440 Hz) and beat (ISI=667 ms), followed by an alternation of eight target and eight distractor tones of variable frequencies (with a s.d. of 0.2 in base-2 logarithmic units) presented in a quasi-rhythmic manner (ISI=667±67 ms). Importantly, targets and distractors occurred in phase and antiphase with the preceding references, respectively, so that participants could use the beat provided by the references to distinguish targets from distractors, which were otherwise perceptually indistinguishable.

An additional distractor tone was inserted in Experiment 2, between the last reference tone and the first target, yielding sequences of 21 pure tones. This extra tone was added so that two distractors surrounded the first target, like for the other targets. This slight modification did not produce any quantifiable effect on human behaviour. The interleaved target and distractor sequences were presented in a rhythmic or quasi-rhythmic manner at 1.5 Hz (ISI=667±0 or 67 ms). The distribution of the 67-ms jitter across the tones of each sequence was approximately Gaussian in Experiments 1 and 3 and uniform in Experiment 2, with the additional constraint of being shorter than 141 ms (to ensure that no overlap between targets and distractors could occur).

Each participant started the experiment with a short training session. During training, the task was made easier by increasing the relative distance between the mean frequency of targets *f*^tar^ and *f*_0_, and by decreasing the volume of distractors. Following this short training session, participants performed a psychophysical staircase with *f*^tar^ as the varying parameter. The staircase was set to obtain 75% of categorization performance in the listen condition (and the fully rhythmic condition in Experiment 2). Each experiment was divided into multiple sessions, each lasting ~60 min. Two hundred ten trials per condition (listen or motor-tracking) were acquired using a blocked design in Experiments 1 and 3, the conditions alternating every 30 trials. One hundred forty-four trials per condition (listen or motor-tracking × 4 jittering conditions) were acquired in Experiment 2, the rhythmicity of targets and/or distractors being randomized at the trial level, and the task (listen or motor-tracking) alternating every 24 trials. Feedback was provided after each trial to indicate correct/incorrect responses, and more general performance feedback indicating the total number of correct responses was given after every 60, 48 and 60 trials, respectively, for motivational purposes.

In the motor-tracking condition, participants were required to follow the beat with their finger on a noiseless laptop pad (Apple MacBook Pro) from the beginning of the sequence (the second reference tone), so that their rhythm was stabilized when the target–distractor sequence started. Participants were asked to stay still in the listen condition, not moving any part of their body, so as to minimize the overt involvement of the motor system in this baseline condition.

### Sensorimotor simultaneity index

To compute the SSI, we first established a theoretical reference beat corresponding to occurrences at 1.5 Hz along the sequence duration (Fig. 1a). We corrected for a possible delay in the recording of the motor acts by extracting the motor-tracking sequence and aligning it to the theoretical reference beat one so as to minimize the trial-averaged delay between the two sequences. While this approach prevents us from studying the absolute asynchrony—or phase—between motor and sensory events^23^, it has the advantage of normalizing the sensorimotor index across participants and provides a reference phase, corresponding to zero-lag, upon which statistical models can be compared. The SSI thus refers to the relative distance between motor and tone occurrences. Importantly, this normalization process is constant within each participant, allowing for comparisons between different SSI values. Occasional absences of motor acts (<2%) were interpolated linearly from the preceding and following ones. We considered the distance between two successive motor acts as one oscillatory cycle and fixed the act as *ϕ*=0.

We indexed the occurrence of each tone with respect to the motor-tracking sequence in radians, with zero corresponding to perfect simultaneity between a tone and the closest motor act. Target and distractor gains were finally sorted according to the SSI, by binning the raw data in 64 overlapping octiles before the multivariate logistic regression. To estimate the SSI corresponding to the maximal sensory gain, we fitted a multivariate logistic regression model including two multiplicative interaction terms corresponding to both the cosine (cos (SSI_*k*_)·*f*_*k*_) and sine (sin (SSI_*k*_)·*f*_*k*_) of the SSI—on the basis of equation 2.

### Statistical procedures

All analyses were performed at the single-subject level and followed by standard parametric two-sided tests at the group level (for example, paired *t*-tests, repeated-measures ANOVA) to assess reliable within-subject differences between conditions. This scheme ensures that between-subject variability in overall motor tracking performance is appropriately controlled for and cannot account for significant group-level effects. Quantitative analyses are based on best-fitting parameter estimates from the logistic regression model. Note that parameter estimates are not bounded and meet the *a priori* assumptions of standard parametric tests.

We complemented standard parametric tests with Bayes Factors in order to distinguish between an insensitive statistical test (not providing evidence in favour or against the null hypothesis) from a genuine absence of difference^57^. For this purpose, we computed a group-level, random-effects Bayes Factor under the exact same assumptions as a standard *t*-test: that the distribution of the observed effect across individuals can be approximated by a normal distribution of the mean *μ* and s.d. *σ*. To compute the Bayes Factor, we computed the maximum log-likelihood of two models: the ‘null’ hypothesis, which assumes that *μ*=0 and has therefore one less parameter than the ‘effect’ hypothesis for which both *μ* and *σ* can be adjusted freely to the observed data. We then used the Akaike Information Criterion^58^ to compare the two models and compute the corresponding Bayes Factor. Note that the maximum attainable evidence in favour of the ‘null’ hypothesis grows with the degrees of freedom of the test (here the number of participants). While Bayesian statistics are usually not considered in terms of thresholds, it is generally assumed that a Bayes factor below 1/3 corresponds to substantial evidence in favour of the ‘null’ hypothesis, whereas a Bayes Factor above 3 corresponds to substantial evidence in favour of the ‘effect’ hypothesis^57^.

## Additional information

**How to cite this article:** Morillon, B. *et al.* Motor contributions to the temporal precision of auditory attention. *Nat. Commun.* 5:5255 doi: 10.1038/ncomms6255 (2014).

## Supplementary Material

Supplementary Information Supplementary Figures 1-4
