# Supplementary material for: Motor contributions to the temporal precision of auditory attention
Source: Nat Commun. 2014 Oct 15;5:5255. doi: 10.1038/ncomms6255 (PMC4199392; doi:10.1038/ncomms6255)
Supplement: Supplementary Information — Supplementary Figures 1-4 [file ncomms6255-s1.pdf]

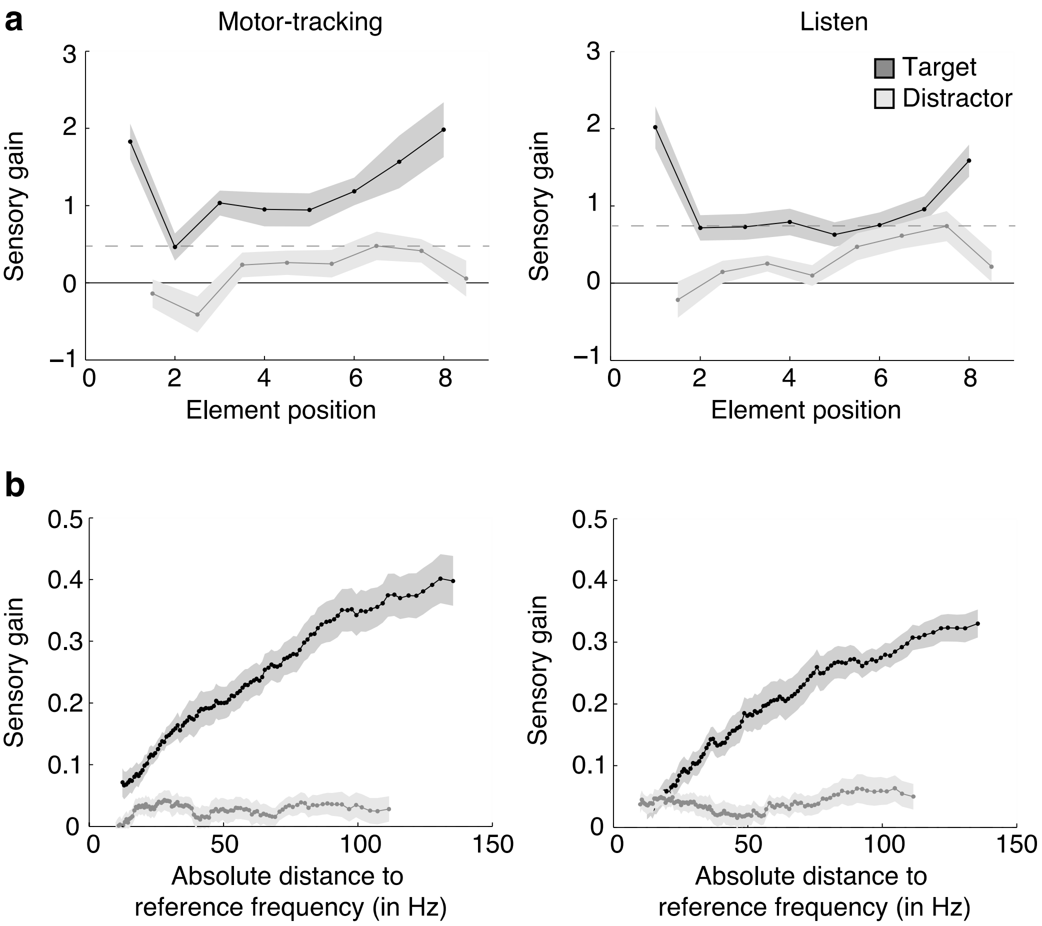

Supplementary Figure 1. Experiment 1. Sensory gain profiles for targets and distractors as a function of (**a**) element position in the sequence and (**b**) absolute distance to the reference frequency, for *motor-tracking* (left panels) and *listen* (right panels) conditions. Shaded error bars indicate s.e.m (n = 21).

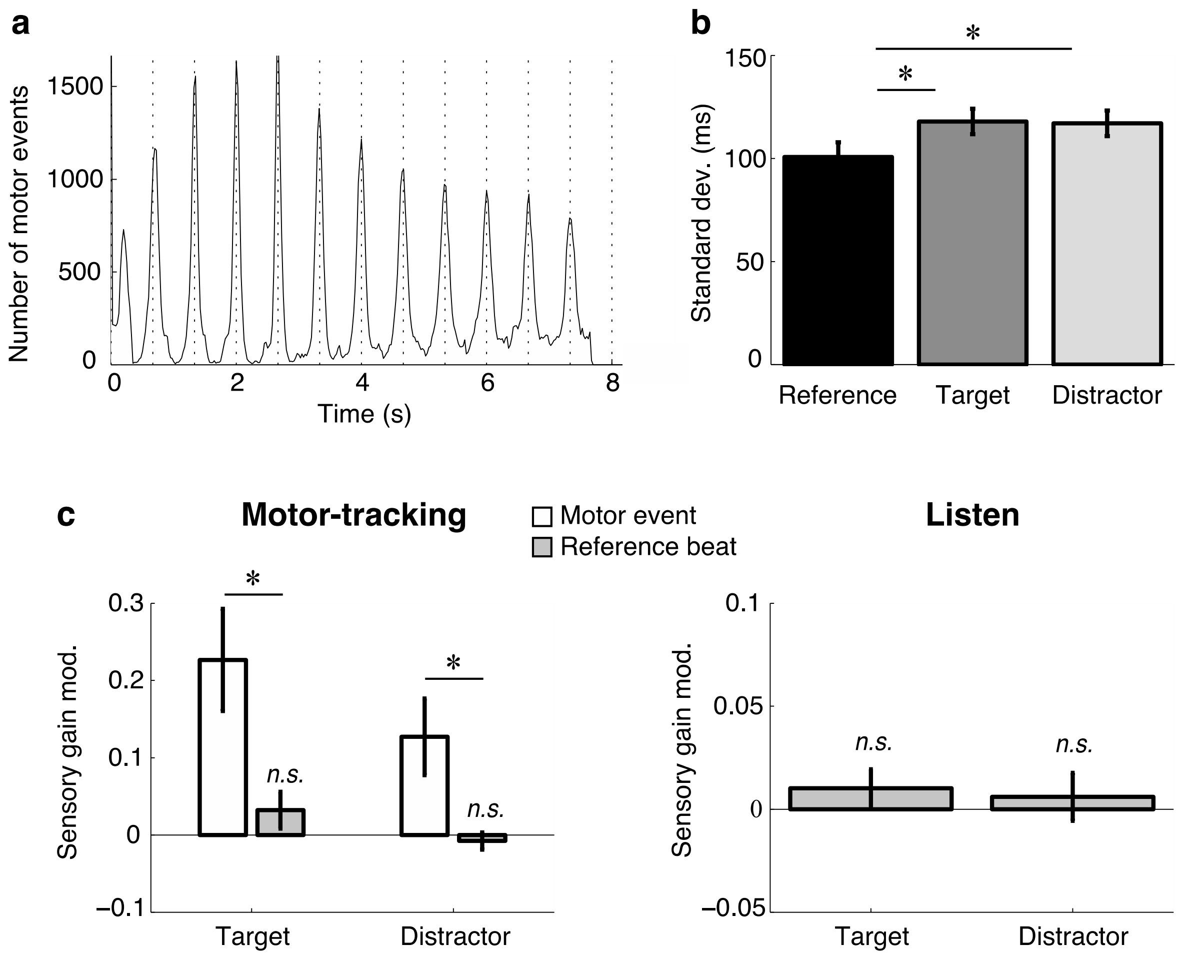

Supplementary Figure 2. Experiment 1. (**a**) Averaged motor acts distribution over time, superimposed with the reference beat (dashed lines). (**b**) Standard deviation of the distribution of the temporal distance between each motor act and the theoretical reference beat (black), targets (dark grey) and distractors (light grey). (**c**) *Left panel:* Amount of behavioral variability explained by taking into account the temporal distance between tones and motor acts (white bars) or reference beat (grey bars) occurrences, in a parametric model linking positive modulation with simultaneity ($\varphi = 0$). *Right panel:* Amount of behavioral variability explained in the *listen* condition by taking into account the temporal distance between tones and reference beat occurrences. Error bars indicate s.e.m. Stars/*n.s.* indicate significant/non-significant differences (n = 21; paired *t* tests or *t* tests against zero; $^*p < 0.05$).

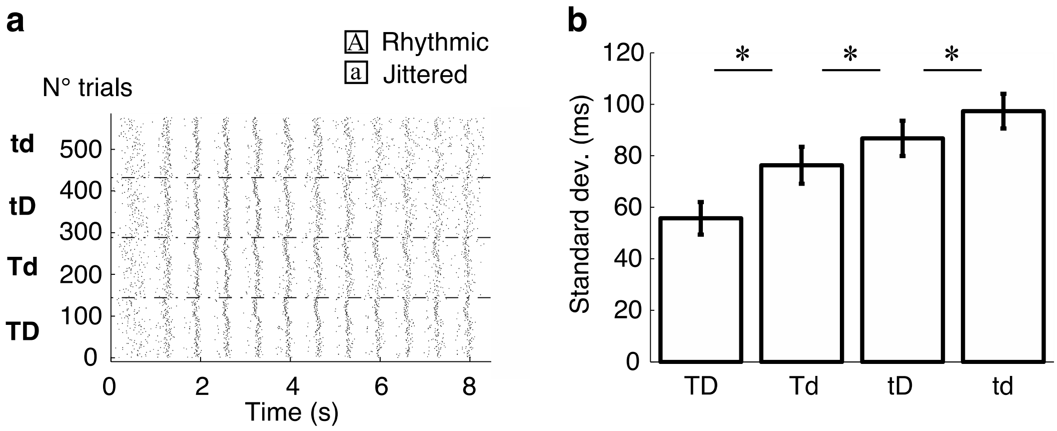

Supplementary Figure 3. Experiment 2. (**a**) Single-trial motor acts distribution over time for one representative participant, sorted per condition. T/t indicates *target* rhythmic/jittered conditions and D/d *distractor* rhythmic/jittered conditions. (**b**) Standard deviation of distribution of the temporal distance between each motor act and the theoretical reference beat, for each condition. Error bars indicate s.e.m. Stars indicate significant differences (n = 18; paired *t* tests; $*p < 0.05$).

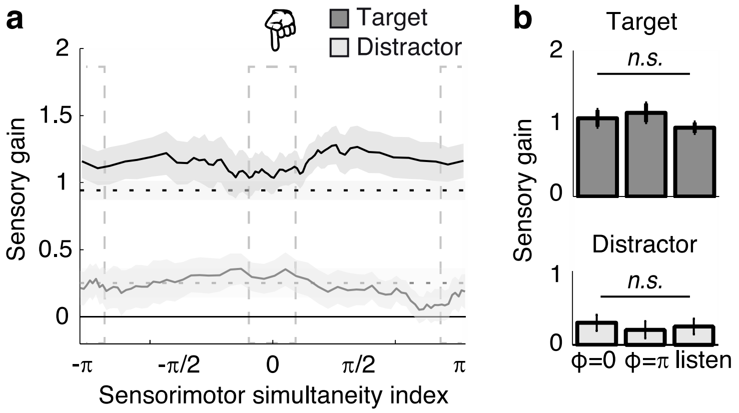

Supplementary Figure 4. Experiment 3. (**a**) Target/distractor gains sorted according to their temporal distance to motor acts (SSI; dashed lines correspond to the *listen* condition). (**b**) Detail of the model-predicted best ($\varphi = 0$) and worst ($\varphi = \pi$) octile, and comparison with the *listen* condition. (Shaded) error bars indicate s.e.m. *N.s.* indicates non-significant differences (n = 21; paired *t* tests; $*p < 0.05$).
